# Protocol for Engineered Compositional Asymmetry Within Nanodiscs

**DOI:** 10.3390/membranes16010044

**Published:** 2026-01-16

**Authors:** Christopher F. Carnahan, Wei He, Yaqing Wang, Matthew A. Coleman, Atul N. Parikh

**Affiliations:** 1 Biophysics Graduate Group, University of California, Davis, CA 95616, USA; cfcarnahan@ucdavis.edu; 2 Biosciences and Biotechnology Division, Lawrence Livermore National Laboratory, Livermore, CA 94550, USA; he4@llnl.gov (W.H.);; 3 Materials Science Division, Lawrence Livermore National Laboratory, Livermore, CA 94550, USA; 4 Department of Biomedical Engineering, University of California, Davis, CA 95616, USA; 5 Institute for Digital Molecular Analytics & Science, Nanyang Technological University, Singapore 639798, Singapore

**Keywords:** lipid, asymmetry, apolipoprotein, cyclodextrin, GUVs, nanodiscs

## Abstract

Membrane proteins remain the most challenging targets for structural characterization, yet their elucidation provides valuable insights into protein function, disease mechanisms, and drug specificity. Structural biology platforms have advanced rapidly in recent years, notably through the development and implementation of nanodiscs—discoidal lipid–protein complexes that encapsulate and solubilize membrane proteins within a controlled, native-like environment. While nanodiscs have become powerful tools for studying membrane proteins, faithfully reconstituting the compositional asymmetry intrinsic to nearly all biological membranes has not yet been achieved. Proper membrane leaflet lipid distribution is critical for accurate protein folding, stability, and insertion. Here, we share a protocol for reconstituting tailored compositional asymmetry within nanodiscs through membrane extraction from giant unilamellar vesicles (GUVs) treated with a leaflet-specific methyl-β-cyclodextrin (mβCD) lipid exchange. Nanodisc asymmetry is verified through a geometric approach: biotin-DPPE-preloaded mβCD engages in lipid exchange with the outer leaflet of POPC GUVs solubilized by the lipid-free membrane scaffold protein (MSP) Δ49ApoA-I to form nanodisc structures. Once isolated, nanodiscs are introduced to the biotin-binding bacterial protein streptavidin. High-speed atomic force microscopy imaging depicts nanodisc–dimer complexes, indicating that biotin-DPPE was successfully reconstituted into a single leaflet of the nanodiscs. This finding outlines the first step toward engineering tailored nanodisc asymmetry and mimicking the native environment of integral proteins—a potentially powerful tool for accurately reconstituting and structurally analyzing integral membrane proteins whose functions are modulated by lipid asymmetry.

## 1. Introduction

Integral membrane proteins are one of the most challenging targets in structural biology. Despite making up ~25% of all proteins, less than 2000 unique structures are solved—less than 2% of the membrane proteins represented in the Protein Data Bank repository. The lack of available structures is a direct result of the historical difficulties in crystallizing membrane proteins in membrane-like environments and characterizing flexible, conformationally diverse nanoscale peptides within their partially hydrophobic environments. Unraveling the complexities of these peptides has been so important that many who develop methods to solve integral protein structures have received Nobel Prizes for their work [1,2,3,4,5].

In recent years, this has changed rapidly. Newer methods, including cryo-electron microscopy [6] and ultrafast X-ray diffraction [7] techniques, are enabling the high-resolution determination of three-dimensional structures of integral proteins. An important wet chemical method that enables the use of these techniques is the use of nanodiscs, a.k.a nanolipoprotein particles (NLPs) —self-assembling discoidal lipid bilayers (8–16 nm in diameter) stabilized by amphipathic α-helical membrane scaffold proteins (MSPs) [8,9]—to reconstitute integral proteins for structural investigation [10,11]. This development in biotechnology has provided an important advantage in deciphering integral protein structure through improvements to isolation, purification, structural resolution, and functional characterizations.

Despite the progress that these developments portend, the reconstitution of many integral proteins within nanodiscs is still difficult. This is due to the behavior of many membrane proteins that orient vectorially with the asymmetric membrane environment, in which mimicking within nanodiscs remains a persistent challenge. Although lipid composition can be tuned during nanodisc self-assembly, replicating the compositional asymmetry of cellular membranes—the unequal distribution of lipid species present between leaflets—has not yet been achieved. Biological membranes are almost universally asymmetric [12,13], with each leaflet distinct in the distribution of lipid acyl chain length, saturation state, head and backbone composition, and chirality. Maintaining asymmetry (through ATP-dependent flippases and free-energy-driven scramblases) is energetically unfavorable and comes at a substantial cost to the cell. However, this is a necessary expenditure, as membrane asymmetry dictates the directionality required for proper integral protein insertion [14], cell–cell signaling [15], folding, and stability [16]. For these reasons, the majority of integral protein structures remain unsolved, with even more experiments unverified, posing a great challenge to structural biologists.

Here, we outline and share the results of a protocol that indicates that compositional asymmetry, engineered via leaflet-specific methyl-β-cyclodextrin (mβCD) lipid exchange in giant unilamellar vesicles (GUVs), can be successfully translated into nanodisc structures stabilized by a truncated isoform of the MSP, apolipoprotein A-1, in which the first 49 amino acids are removed (Δ49ApoA-I). With a robust approach, the ability to synthesize nanodiscs with defined leaflet asymmetry represents a significant advance. Such tailor-made nanodiscs could become a powerful tool for accurately reconstituting and structurally analyzing integral membrane proteins whose functions are modulated by lipid asymmetry—expanding the experimental toolkit for membrane biology, biophysics, and structural biochemistry.

## 2. Overview of the Procedure

**Figure 1:**
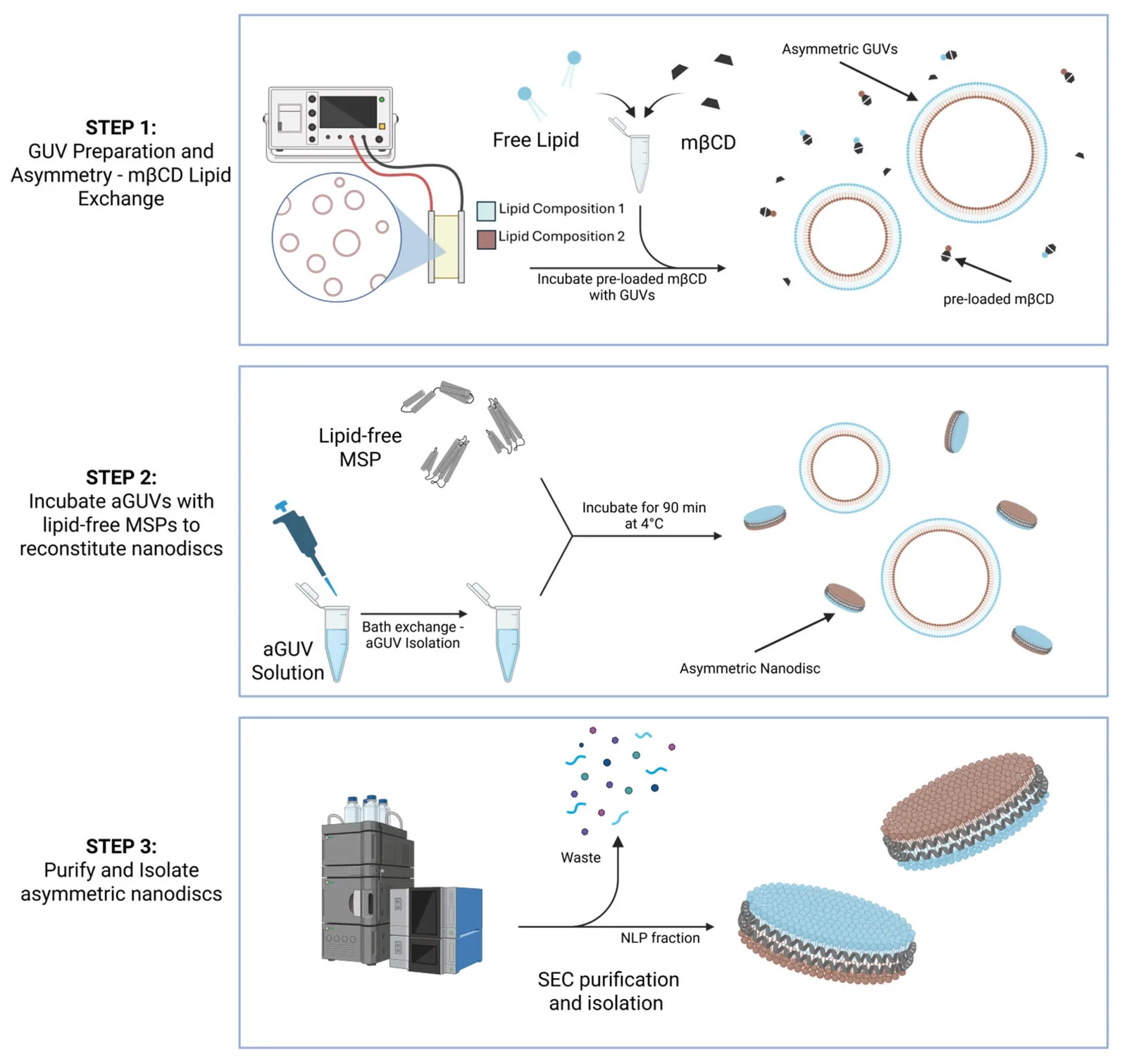
Experimental workflow for the preparation of compositionally asymmetric nanodiscs in three stages: (**1**) mβCD leaflet exchange with giant unilamellar vesicles, (**2**) MSP incubation with isolated aGUVs, and (**3**) size exclusion chromatography (SEC) purification and separation of the nanodisc fraction.

The preparation of nanodisc asymmetry consists of three main stages (Figure 1), which are described here as a generalized concept.

### 2.1. Part 1: mβCD-Mediated Lipid Asymmetry in GUVs

A collection of GUVs are synthesized using the well-established electroformation technique (see Methods) from a lipid shock composed of the first desired leaflet distribution. An aliquot of the GUV solution is incubated with mβCD preloaded with the second desired leaflet lipid composition: lipid exchange occurs between the preloaded mβCD and the outer leaflet of the GUVs.

### 2.2. Part 2: Δ49ApoA-I Incubation and Nanodisc Reconstitution

The preparation of nanodiscs from isolated asymmetric GUVs involves incubation with lipid-free Δ49ApoA-I. These MSPs spontaneously dissolve the vesicles to form discoidal complexes of membrane lipids suspended by Δ49ApoA-I.

### 2.3. Part 3: SEC Purification and Isolation of Asymmetric Nanodiscs

Asymmetric nanodiscs were purified using SEC to homogeneity from the solution from part 2. The purified nanodiscs showed diameters averaging 18.7 nm.

## 3. Experimental Design

Methyl-β-cyclodextrin (mβCD) is class of oligosaccharide composed of cyclically bound D-glucose molecules. This molecule’s unique structure produces a particle capable of solubilization in aqueous environments despite housing a hydrophobic cavity within its lumen. This non-polar cavity has been widely used as a means of solubilizing various hydrophobic molecules—namely, sterols [17]—and at high concentrations, mβCD is observed to bind and solubilize lipids [18], modifying the composition of a membrane through a lipidic exchange with individual leaflets within the bilayer [19]. The observation of this behavior led researchers (such as Erwin Londen et al.) to utilize cyclodextrin in a creative way by modulating the lipid composition of a membrane’s extracellular leaflet to create tailored compositional asymmetry in synthetic vesicles [20,21]. Compositional asymmetry generated in this way is relatively stable as a result of minimal phospholipid transversal diffusion (flip-flop, 10^−15^ s^−1^ [22]). Here, mβCD is utilized in the same manner to both verify and modify the preparation of membrane asymmetry in GUVs.

**Figure 2:**
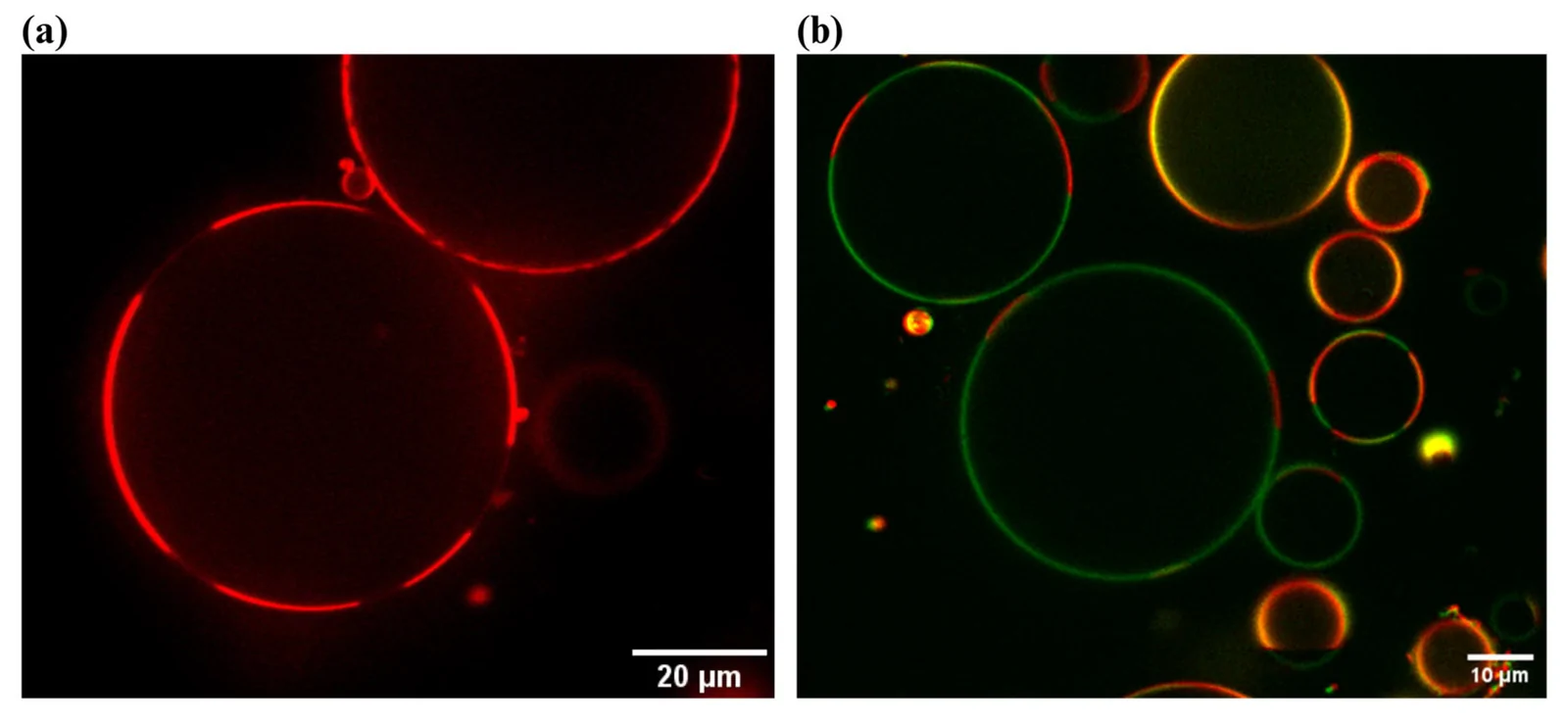
(**a**) Confocal xy-scan of POPC GUVs doped with 3 mol% of Rho-PE (red) following treatment with 0.25 mM mβCD preloaded (3:1 = CD/lipid) with a head-labeled biotinylated DPPE. The reasons for the appearance of domains depleted of rhodamine label are unknown, and not the focus of this manuscript. (**b**) After ~30 min of observation, these GUVs were treated with 0.05 mg/mL FITC–streptavidin (green), labeling complementary regions of the GUV labeled with Rho-PE, indicating binding to biotinylated PE lipids deposited into the vesicular membrane through mβCD lipid exchange.

A control experiment was conducted to verify mβCD’s ability to exchange certain lipids with synthetic GUVs. In this experiment, 2 μL of a palmitoyl-2-oleoyl-sn-glycero-3-phosphocholine (POPC) GUV solution (encapsulated 100 mM sucrose) was diluted in 97.5 μL of 100 mM glucose along with 0.5 μL of 50 mM mβCD preloaded (3:1 = mβCD/lipid) with head-labeled 1,2-dipalmitoyl-sn-glycero-3-phosphoethanolamine-N-(biotinyl) (biotinylated DPPE). The reaction was halted after 1 min by dilution of the surrounding bath with 100 mM glucose (the higher density of encapsulated 100 mM sucrose reduces the loss of vesicles). Halting the reaction is required, as lipid removal by free mβCD will induce a differential stress across the membrane, resulting in deformation. The GUVs prepared this way remained stable throughout an observation period of ~30 min. Interestingly, once incubated, POPC GUVs were observed to exhibit phase domains of complementary membrane arcs of labeled and unlabeled membrane domains. The reason for this separation is unknown (potentially related to the DPPE-biotinylated headgroups) and is not the focus of this report (Figure 2a). Following this period of observation, 0.05 mg/mL of a FITC-labeled (green) streptavidin (SA) was introduced into the GUV solution (Figure 2b). We observed a heightened FITC-labeled SA fluorescence signal at the membrane’s surface, indicating that the lipid exchange of biotin-PE was successful.

**Figure 3:**
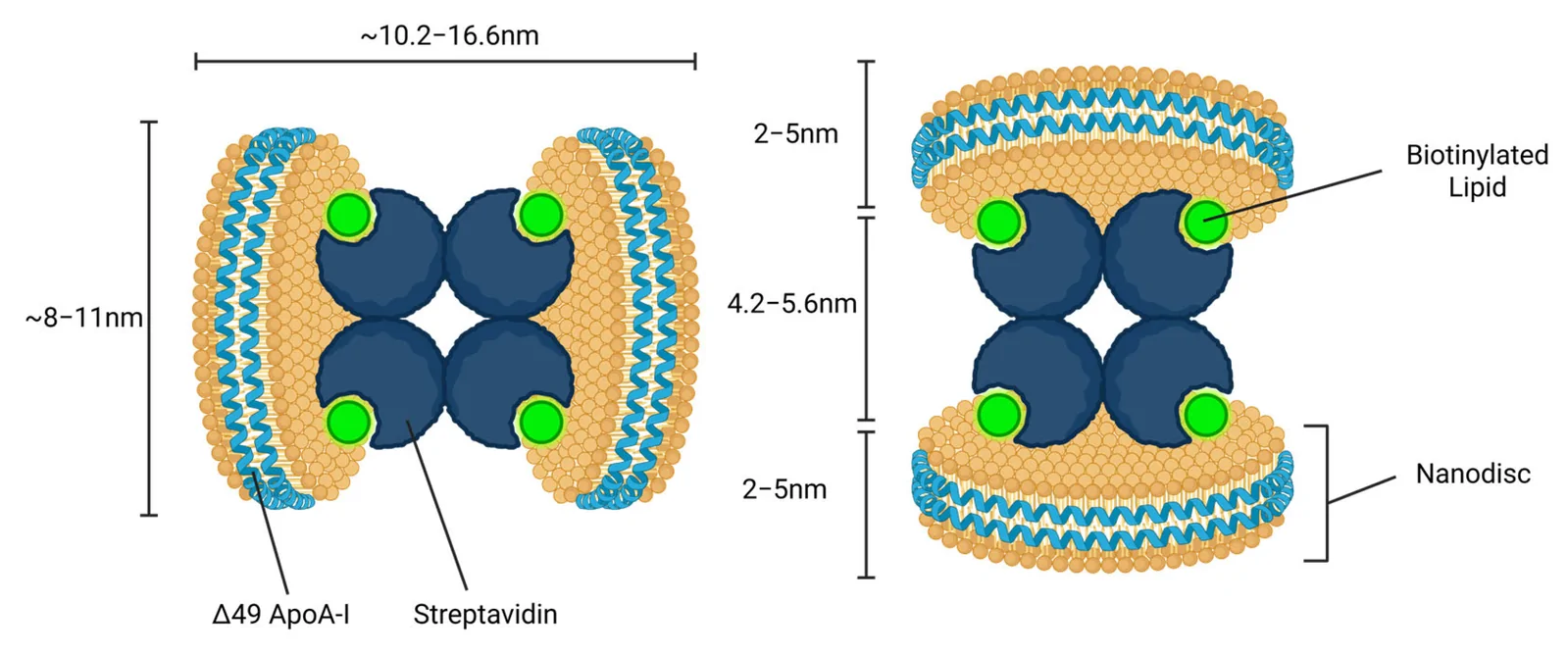
Diagram of the expected geometry of streptavidin-bound nHDL dimer complex. Nanodiscs (yellow and light blue) with engineered, asymmetric incorporation of biotinylated PE lipids (green) into a single leaflet of the bilayer will form a dimer structure when bound to the tetrameric structure of streptavidin’s (dark blue) binding sites.

Characterizing the membrane asymmetry of a synthetic vesicle is exceptionally difficult, often requiring highly advanced techniques, including combinations of NMR [23], GC-MS [24], and HPTLC [25] or lipid-specific approaches with zeta potential of negatively charged lipids or TMA-DPH. Furthermore, the extent to which MSP-mediated solubilization results in lipid redistribution or alters transversal diffusion energetics is unknown. Any lipid flip-flop during the formation of nanodiscs will alter the asymmetry produced in mother GUVs by mβCD activity. To characterize leaflet asymmetry within nanodiscs and determine whether lipid redistribution occurs during MSP-mediated solubilization, we developed a novel and innovative approach. Nanodiscs were formed from asymmetric GUVs synthesized through an mβCD-mediated leaflet exchange of headgroup-labeled biotinylated DPPE lipids. Once isolated, the nanodiscs were treated with the biotin-binding bacterial protein streptavidin (SA); the interaction between biotin and SA is one of the strongest non-covalent instances of binding found in nature, with an association constant of ~10^15^ [26]. Utilizing high-speed atomic force microscopy (HS-AFM), the nanodiscs were imaged, with the following potential outcomes. (1) Single discoidal complexes are resolved that resemble the control experiment (without added SA), indicating that biotin-PE was not adequately deposited into the GUV membranes or incorporated by the MSPs. (2) Discoidal complexes are bound from both ends forming a chain-like structure. This would indicate that biotin-PE was incorporated into the nanodiscs; however, asymmetry was not maintained, and SA bound them from both sides. Finally, (3) nanodiscs are imaged as dimer complexes, indicating that biotin-PE asymmetry was maintained and SA bound only one of each face of the nanodisc (Figure 3).

## 4. Materials

### 4.1. Reagents

#### 4.1.1. Giant Unilamellar Vesicles

- Palmitoyl-2-oleoyl-sn-glycero-3-phosphocholine (POPC) (Avanti Polar Lipids, Alabaster, AL, USA).
- Lissamine rhodamine B 1,2-dioleyl-sn-glycero-3-phosphoethanolamine (Rho-B DOPE) Avanti Polar Lipids, Alabaster, AL, USA.
- 1 mL of 100 mM sucrose.
- 1 mL of 100 mM glucose.
- CHCl_3_ (chloroform) (Fisher Scientific, Waltham, MA, USA).

#### 4.1.2. mβCD–Biotin Complex

- 1,2-dipalmitoyl-sn-glycero-3-phosphoethanolamine-N-(biotinyl) (sodium salt) (biotin-PE) (Avanti Polar Lipids).
- Methyl-β-cyclodextrin (mβCD) (powder) (SigmaAldrich, St Louis, MO, USA).
- CH_3_OH (methanol) (Fisher Scientific).

#### 4.1.3. Cell-Free Δ49ApoA-I Protein

- RTS 500 ProteoMas-ter E. coli HY Kit (Biotechrabbit GmbH, Hannover, Germany).
- 15 µg Δ49ApoA-I plasmid DNA (Promega, Madison, WI, USA).
- 20 µL Fluoro-Tect™ GreenLys tRNA (Promega, Madison, WI, USA).
- 10 mM imidazole (Sigma-Aldrich, St Louis, MO, USA).
- Equilibration buffer (50 mM NaH_2_PO_4_, 300 mM NaCl, pH 8.0).
- 6 × 300 µL elution buffer (50 mM NaH_2_PO_4_, 300 mM NaCl, 250 mM imidazole, pH 8.0).

### 4.2. Equipment

- Glass-bottom, 96-well plates (Cellvis, Mountian View, CA, USA).
- 2 x Indium tin oxide (ITO)-coated glass slides (5–25 Ω) (Delta Technologies, Ltd., Rochester Hills, MI, USA).
- Manual Teflon-tipped syringes (Agilent Technologies, Santa Clara, CA, USA).
- 1 mm thick rubber O ring (Ace Hardware, Davis, CA, USA).
- High-vacuum grease (Dow Corning, Auburn, MI, USA).
- AFG3022B Dual Channel Arbitrary/Function Generator 25 MHz 250 MS/s (Tektronix, Beaverton, OR, USA).
- SEC (Superdex 200 Increase 10/300 GL column, GE Healthcare, Chicago, IL, USA).

## 5. Procedure

### 5.1. Preparation of Giant Unilamellar Vesicles

GUV preparation via electroformation is a well-established process [27,28]. A lipid stock of a desired composition at a concentration of 2 mg/mL is prepared in chloroform. For single-component synthetic membranes, POPC (99 mol %) is dissolved with a Rho-B DOPE label (1 mol %). Then, 15 µL is deposited onto the conductive side of two ITO-coated slides and spread evenly on half of the slide as it evaporates. These slides are further dried inside a vacuum desiccator anywhere from 2 to 24 h. Once dry, a chamber is formed using a 1 mm thick rubber O ring (Ace Hardware, Davis, CA, USA) and sealed with high-vacuum grease (Dow Corning, Midland, MI, USA). After the O ring is adhered to the conductive side of the ITO slide, the chamber is hydrated with ~1 mL of a 100 mM sucrose solution. The chamber is sealed with the second, inward-facing lipid cake, ITO slide ensuring no trapped air bubbles. The two slides form the chamber in a way that the halves without dried lipid are facing in opposite directions to avoid the alligator clips from the function generator from touching both slides, effectively forming a less resistant pathway for any current.

A 2.2 V(pp) AC Sine wave is applied across the two slides at a frequency of 10 Hz for 1 h, followed by another 2.2 V(pp) AC Sine wave at a frequency of 2 Hz for 30 min. Samples undergo this step covered to protect from light. After GUV formation, the chamber is disassembled, and the solution is pipetted into a small centrifuge tube and stored at 4 °C. The vesicles prepared are either used or discarded within a week of preparation.

### 5.2. Preparation of mβCD–Biotin-DPPE Complex Solution

In this step, 20 ul of 50 mM 3:1 mβCD–biotin-DPPE is prepared by dissolving 2.6 mg of mβCD and 25.1 uL of 25 mg/mL 1,2-dipalmitoyl-sn-glycero-3-phosphoethanolamine-N-(biotinyl) (biotin-PE) in methanol. Once fully dissolved, the solution is evaporated with nitrogen and placed in a desiccation chamber overnight. The film is rehydrated with 58.2 uL DI to bring the final concentration to 50 mM and sonicated for 5 min. The final should be slightly turbid and stored at 4 °C.

### 5.3. Cell-Free Synthesis of Δ49ApoA-I Protein

Cell-free reactions were set up using an RTS 500 ProteoMaster E. coli HY Kit (Biotechrabbit GmbH, Hanover, Germany). Reaction components (lysate, reaction mix, feeding mix, amino acid mix, and methionine) were combined as specified by the manufacturer. Each 1 mL reaction contained 15 µg Δ49ApoA-I plasmid DNA and 20 µL Fluoro-Tect™ GreenLys tRNA (Promega, Madison, WI, USA). The reactions were incubated at 30 °C, with shaking at 300 rpm for 18 h in a floor shaker. After the reaction, ApoA-I protein was purified using nickel affinity chromatography. Briefly, 1 mL of 50% slurry complete His-Tag Purification Resin (Roche Molecular Diagnostics, Basel, Switzerland) was equilibrated with equilibration buffer (50 mM NaH_2_PO_4_, 300 mM NaCl, pH 8.0) with 10 mM imidazole (Sigma-Aldrich, St Louis, MO, USA) in a 10 mL chromatography column. The total cell-free reaction was mixed with the equilibrated resin and was incubated/nutated at 4 °C for 1 h. The column was then washed 6 times with 1 mL wash buffer containing 50 mM NaH_2_PO_4_, 300 mM NaCl, and 20 mM imidazole, pH 8.0. ApoA-I was eluted with 6 × 300 µL elution buffer (50 mM NaH_2_PO_4_, 300 mM NaCl, 250 mM imidazole, pH 8.0). All elutions were analyzed by SDS-PAGE and peak fractions containing labeled protein were combined. Pooled fractions were dialyzed in PBS (pH 7.4) and then stored at 4 °C. Protein levels were quantified using a Qubit protein test according to the manufacturer’s instructions (Thermo Fisher Scientific, Carlsbad, CA, USA).

### 5.4. mβCD–Biotin-Mediated Leaflet Exchange in Giant Unilamellar Vesicles

Here, 2 µL of POPC GUV solution was incubated with 0.5 µL of 50 mM (0.25 mM final concentration) mβCD–biotin-DPPE complex solution in 97.5 µL of 100 mM glucose (Figure 2a). GUVs were incubated with this mβCD–biotin-PE complex for 1 min before slowing the interaction via bath dilution, as explained in the following section.

### 5.5. Reconstitution of Nanodiscs from Asymmetric Giant Unilamellar Vesicles

GUVs incubated with 0.25 mM biotin-PE-loaded mβCD (3:1 = CD/biotin-PE) were isolated through an isomolar bath exchange (GUVs encapsulate 100 mM sucrose, rendering them higher density than the 100 mM glucose bath, therefore separating from the bulk solution). Gently, so as to not disturb settled vesicles, 25 µL of 100 mM glucose was added; there was a 10 s wait, and the 25 µL of the solution was removed. This was repeated six times. The mβCD concentration was now less than 50 µM, low enough to not induce membrane deformations. Then, 100 µL of the vesicle solution was solubilized by adding 1.75 µL of 15 mg/mL Δ49ApoA-I and incubating for 1.5 h at 4 °C.

### 5.6. SEC Isolation of Asymmetric Nanodiscs

**Figure 4:**
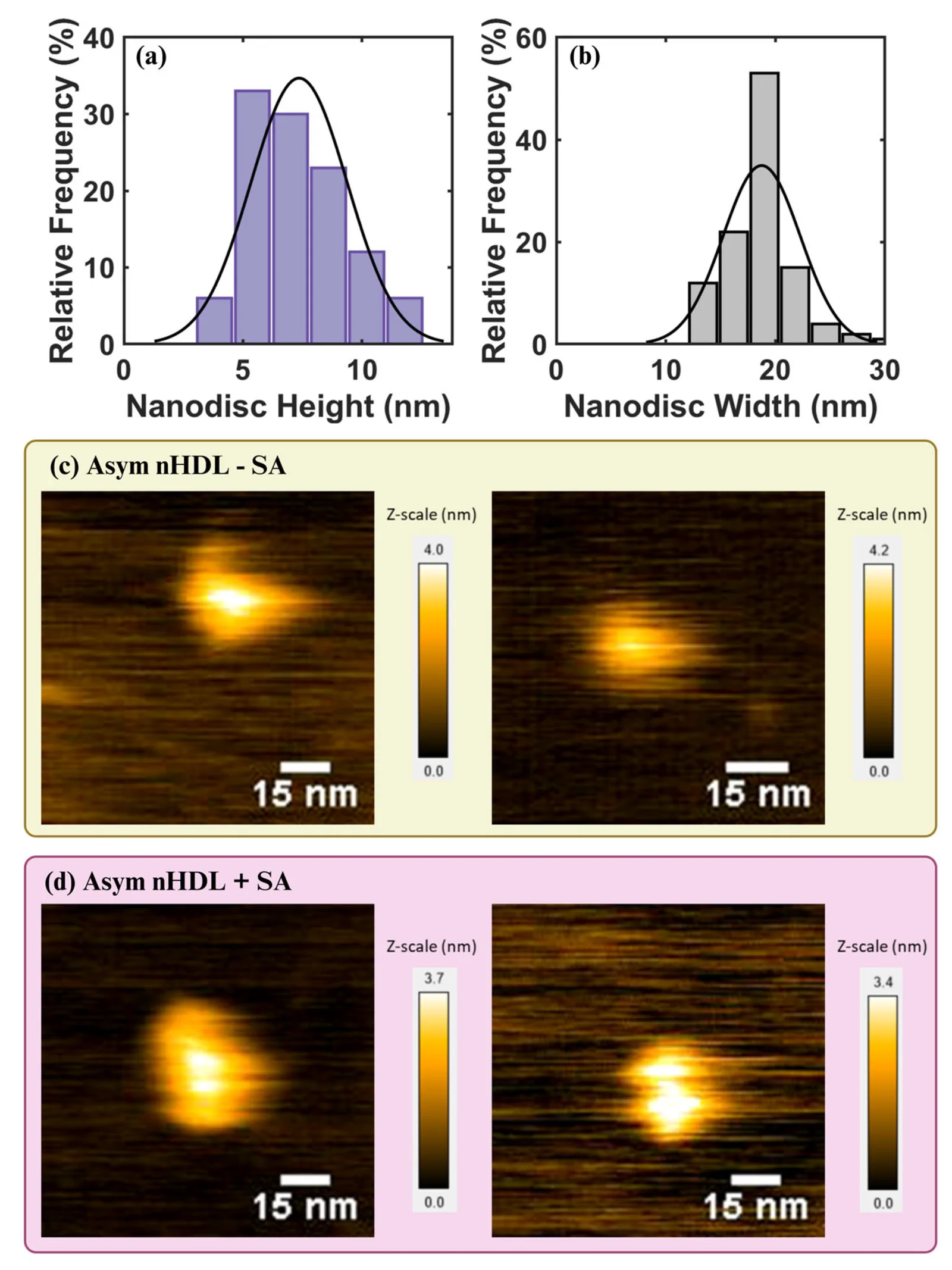
Relative histogram of discoidal structures—(**a**) height averaging 7.3 ± 2.0 nm and (**b**) width averaging 18.7 ± 3.5 nm—formed and isolated from GUVs incubated with 1 mM biotin-PE-loaded mβCD (3:1 = CD/biotin-PE). (**c**) AFM images depicting single nanodisc appearance, and (**d**) AFM images following the addition of 2.16 µM of SA (~1/4 molar ratio with Δ49ApoA-I) depicting nanodisc–dimer complexes.

Asymmetric nanodiscs were purified by SEC (Superdex 200 Increase 10/300 GL column, GE Healthcare, Chicago, IL, USA) with a flow rate of 0.5 mL/min to ensure no overlap in the elution of disassembled Δ49ApoA-I and intact nanodiscs. The nanodiscs were purified to homogeneity with diameters averaging 18.7 ± 3.5 nm (Figure 4b).

## 6. Anticipated Results

### 6.1. High-Speed AFM Characterization of Reactant Nanodiscs

A high-speed atomic force microscope (RIBM, Tsukuba, Japan) was equipped with a small cantilever (Ultra-Short Cantilevers (USC, NanoWorld, Neuchâtel, Switzerland): spring constant, k = 0.15 N/m, resonance frequency, f = 1200 kHz in water) and was operated in tapping mode at room temperature. The free oscillation amplitude was 0.9~1.3 nm, and the typical set-point amplitude was 85% of the free oscillation amplitude. For the sample stage, a freshly cleaved mica disc with a diameter of 1.5 mm was fixed on a glass rod with a diameter of 1.5 mm and a height of 2 mm using epoxy glue. The SEC-purified nanodisc fraction was diluted 20× in PBS buffer, and 2 uL of the diluted solution was deposited onto the freshly cleaved mica and incubated for 10 min. The scanner was then mounted above the sample chamber with the cantilever immersed in PBS buffer.

We found single discoidal structures with a height of 7.3 ± 2.0 nm and width of 18.7 ± 3.5 nm (Figure 4a,b)—in excellent agreement with the formation of nanodiscs (Figure 4c) [29,30]—that did not resemble unlipidated protein aggregates [31,32]. HS-AFM images were processed using ImageJ, (https://imagej.net/ij/, accessed on 28 October 2025), (National Institutes of Health, Bethesda, MD, USA). The height and width dimensions of each nanodisc were analyzed with Gwyddion software [33] and the data was plotted with Origin (https://www.originlab.com/).

### 6.2. Characterization of Nanodisc Asymmetry

The SEC-isolated nanodisc solution was incubated with 2.16 µM of SA (~1/4 molar ratio with Δ49ApoA-I) for 30 min at 4 °C before imaging with HS-AFM. Structures imaged from this solution appeared to have a dimer structure (Figure 4d). The nanodisc structures appeared as dimer complexes, indicating that biotin-PE asymmetry was maintained and SA bound only one of each face of the nanodisc. Notably, the dimensions of these structures are not what was predicted in Figure 3. This is likely due to the low lipid/Δ49ApoA-I ratio in this experiment, in which scaffold proteins form a saddle-shaped double belt structure to accommodate smaller lipid concentrations [34]. In this model, lipids may point outward; bound together by SA, the observed dimer structures are consistent.

The appearance of nanodisc–dimer complexes indicates that compositional asymmetry engineered by the leaflet-specific mβCD lipid exchange of biotin-PE lipids in GUVs is maintained in the formation of nanodisc structures by Δ49ApoA-I. To confirm these results, further experiments are required and limitations to the technique must be defined. The next steps should consider alternate mβCD donor and GUV acceptor lipid compositions, and adjustments to the lipid/Δ49ApoA-I ratio. An analysis of nanodisc lipid composition through mass spectrometry could be used to quantify lipid exchange efficiency. A supporting experiment to quantify leaflet asymmetry should be conducted; for example, if the mβCD donor lipid was a negatively charged species, a zeta potential measurement would distinguish differences between leaflet composition through electrostatic interactions. A robust method for the synthesis of tailor-made leaflet asymmetry incorporated into nanodisc particles would serve as a new tool for the reconstitution of compositionally dependent integral membrane proteins for structural analysis.

## Data Availability

The original contributions presented in this study are included in the article. Further inquiries can be directed to the corresponding author.
